# Chemical exfoliation of layered Al_5_C_3_N for the synthesis of AlN nanosheets

**DOI:** 10.1038/s43246-025-01019-3

**Published:** 2025-12-08

**Authors:** Nima Amousa, Melina Poll, Louis Godeffroy, Pedro Berastegui, Norbert H. Nickel, Namrata Sharma, Olivier Donzel-Gargand, Thomas Dittrich, Steffen Fengler, Sebastian Wintz, Tristan Petit, Ulf Jansson, Jesus Gonzalez-Julian

**Affiliations:** 1 Nanoscale Solid−Liquid Interfaces, Helmholtz-Zentrum Berlin für Materialien und Energie GmbH, Berlin, Germany; 2 Institute of Energy Materials and Devices (IMD-2)—Materials Synthesis and Processing, Forschungszentrum Jülich GmbH, Jülich, Germany; 3 Department of Chemistry-Ångström, Uppsala University Box 538, Uppsala, Sweden; 4 Faculty of Mathematics and Natural Sciences, TU Berlin, Berlin, Germany; 5 Department of Material Science and Engineering, Ångström laboratory, Uppsala University, Uppsala, Sweden; 6 Freiberg Instrument GmbH, Freiberg, Germany; 7 Institut für Nanospektroskopie, Helmholtz-Zentrum Berlin für Materialien und Energie GmbH, Berlin, Germany; 8 Laboratory of Thermo-Structural Composites (LCTS), Pessac, France

**Keywords:** Synthesis and processing, Two-dimensional materials

## Abstract

Two-dimensional (2D) aluminum nitride (AlN) represents a promising material with unique properties predicted by density functional theory (DFT), characterized by a honeycomb lattice where Al and N atoms exhibit threefold in-plane coordination. However, the synthesis of free-standing AlN nanosheets has been challenging due to the crystal configurations of the well-known bulk AlN, which presents a hexagonal wurtzite structure with a tetrahedral coordination, preventing its exfoliation to obtain nanosheets. Herein, we propose a facile method involving the preparation of layered-structured aluminum carbonitrides, Al_5_C_3_N, followed by exfoliation into AlN nanosheets, offering a potential route for producing 2D AlN. The Al_5_C_3_N precursor was chemically etched in hydrofluoric acid (HF), breaking the Al-C bonds and exposing the AlN nanosheets. The development of this synthesis method opens up opportunities towards the preparation of 2D AlN and the investigation of its unique properties for applications in sensors and microelectronics.

## Abstract (web summary)

Two-dimensional aluminum nitride holds promise for advanced applications, yet its synthesis is hindered by the bulk material’s hexagonal wurtzite structure which prevents facile exfoliation. Here, the authors present a method using layered-structured aluminum carbonitrides as precursors and hydrofluoric acid chemical etching to produce AlN nanosheets, paving the way for innovations in sensors and microelectronics.

Low-dimensional materials have been found to possess distinct properties from their bulk counterparts^1^. 2D materials, in particular, have garnered significant attention due to their remarkable physicochemical and electrical properties thanks to their high surface-to-volume ratio^1–3^. Graphene was the first 2D material to be discovered in 2004^1^, followed by the development of other compositions containing a single element like silicene^4^ and phosphorene^5^, or several elements, such as hexagonal boron nitride (h-BN)^6^, transition metal dichalcogenides (TMDs)^7^, and transition metal carbides and nitrides (MXenes)^8^. The exploration of novel 2D materials, from other groups of the periodic table, including group-III nitride semiconductor materials, particularly AlN, promises enhanced properties and would broaden their applications in electronic devices, sensors, and optoelectronics^9^.

2D AlN is predicted by DFT calculations, with aluminum and nitrogen atoms that are threefold coordinated in-plane^10,11^. The electronegativity difference between Al and N creates a dipole moment, influencing bond features and properties like a wide band gap (>2.8 eV) and strong CO_2_ adsorption (0.91 eV)^12–14^. DFT calculations have put 2D AlN in the spotlight, but the existing methods, such as chemical vapor deposition (CVD) and molecular beam epitaxy (MBE), suffer from difficulties in controlling thickness and uniformity^15,16^. This problem is related to the two stable configurations of the AlN system, the predicted 2D and the bulk (3D) AlN crystal structure. Bulk-AlN presents a hexagonal wurtzite crystal structure, where the Al and N atoms are tetrahedrally coordinated. The fourfold coordination entails a 3D structure, where all the bonds exhibit the same strength and chemistry in the three axes, excluding the possibility of breaking some specific bonds to obtain a 2D structure. Therefore, alternative strategies need to be developed for the preparation of a 2D structure, where AlN is not the precursor.

An alternative approach in synthesizing 2D materials is preparing layer-structured precursors and, in sequence, exfoliating them into 2D nanosheets, with mechanical exfoliation proving effective for nanolayers held by Van der Waals forces (e.g., graphene), and chemical exfoliation being necessary for strongly bonded nanolayers, as seen in MXenes synthesized from MAX phases^8,17^. Therefore, the preparation of novel layer-structured materials has the potential to allow the preparation of 2D group-III nitrides.

Aluminum carbonitrides are a family of nanolayered materials with the general formula (AlN)_n_Al_4_C_3_, where *n* ranges from one to four^18^. Their crystal structure is arranged in stacking sequences of n[AlN], [Al_2_C_2_], and [Al_2_C] layers. In the case of Al_5_C_3_N (*n* = 1), the stacking sequence corresponds to Al_2_C_2_-AlN-Al_2_C (Fig. 1a), where the AlN layer exhibits a threefold coordination in-plane, with the Al and N atoms arranged in a honeycomb lattice. This in-plane AlN layer is bonded to the carbide layers by a fourth bond between the Al and C in the vertical axis, and the chemical strength of the Al-N bond is stronger than the Al-C bond^19^. Aluminum carbonitrides were characterized in the 1960s, although Al_5_C_3_N was reported in 1935, using small single-crystals that were formed on the walls of a vessel by heating AlN in a carbon crucible at 2000 °C under N_2_ atmosphere^18,20^. Later studies investigated the electronic structure and chemical bonding of Al_5_C_3_N by ab initio calculations^19^, and the synthesis of Al_5_C_3_N containing some Al_6_C_3_N_2_^21^. Structurally, Al₅C₃N consists of alternating strong Al–N layers and weaker Al–C layers, a feature that closely parallels MAX phases, where selective removal of weakly bonded layers led to the discovery of MXenes. Inspired by this analogy, we considered Al₅C₃N as a precursor for producing AlN nanosheets by selectively etching the carbide layers.

**Fig. 1:**
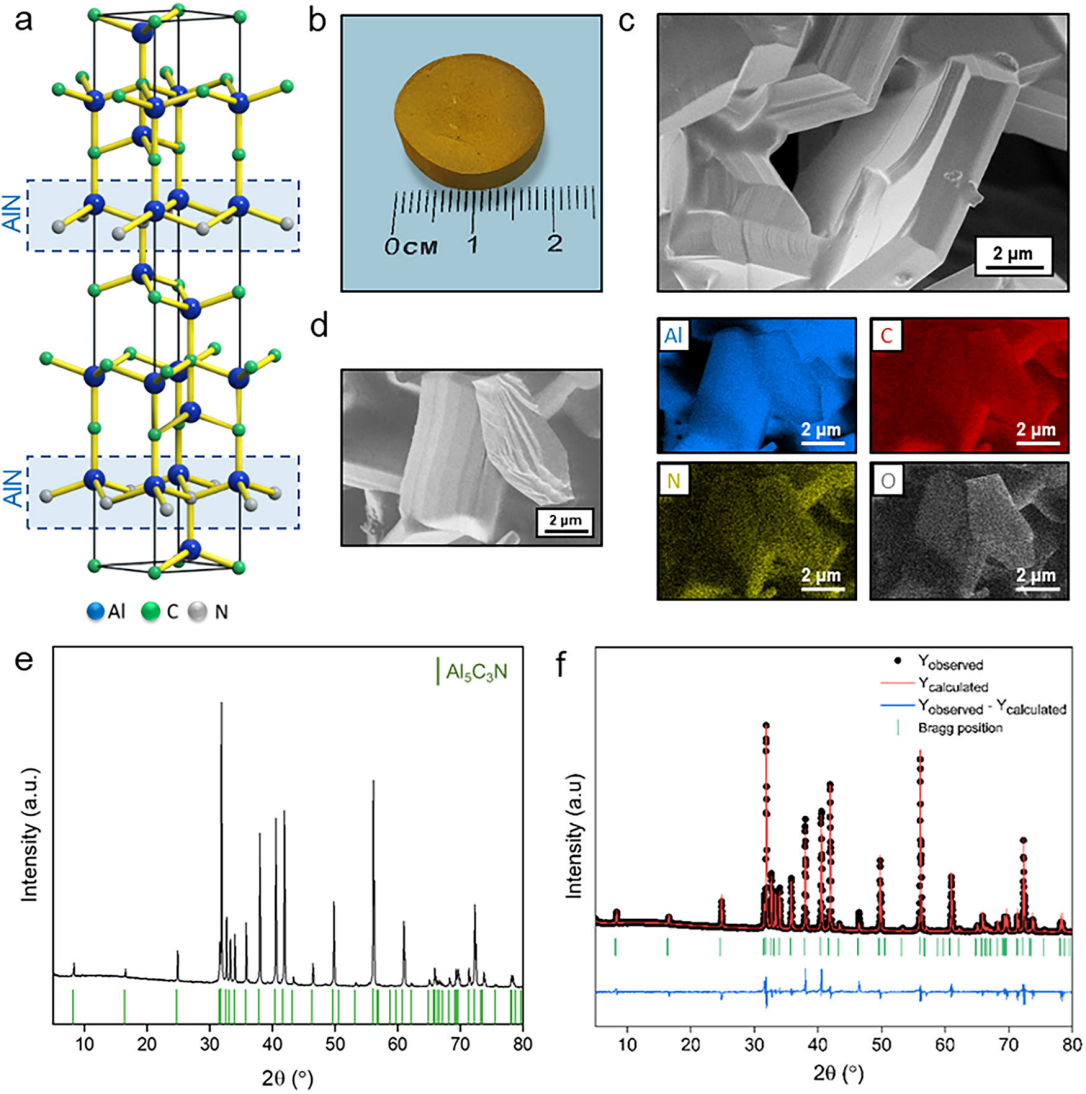
Structural and morphological characterization of Al₅C₃N. **a** Graphical atomic arrangement. **b** Digital image. **c** SEM image. **d** Elemental mapping. **e** XRD pattern. **f** Rietveld refinement.

In this work, we introduce the synthesis of AlN nanosheets by chemical exfoliation of Al_5_C_3_N. Al_5_C_3_N powder was first prepared by utilizing Field-Assisted Sintering Technology/Spark Plasma Sintering (FAST/SPS) of Al_4_C_3_ and AlN powders. Then, a chemical etching strategy, similar to the one used for the MXene synthesis route from MAX phases^8^, was employed to prepare AlN nanosheets from Al_5_C_3_N, inspired by the chemical and structural similarities between MAX phases and aluminum carbonitrides. This study introduces a chemical exfoliation approach for synthesizing multilayered AlN nanosheets from Al₅C₃N. The resulting structures are not monolayer AlN, but they represent an essential first step toward the realization of 2D AlN.

## Results and discussion

### Synthesis and characterization of Al₅C₃N

The resulting sintered Al_5_C_3_N sample is shown in Fig. 1b, displaying its distinct yellow color. The distinct yellow color of the sintered Al₅C₃N pellet is consistent with earlier reports of this phase^20^, and arises from its narrow band gap (0.81 eV^19^). X-ray diffraction (XRD), scanning electron microscopy (SEM), and energy-dispersive X-ray spectroscopy (EDS) confirm the successful synthesis of the layer-structured Al_5_C_3_N precursor. The SEM analysis reveals the distinct layered morphology of Al_5_C_3_N (Fig. 1c), while EDS mapping confirms the presence of Al, C, and N, with additional oxygen detected (Fig. 1d), which is attributed to the native oxide layer of the starting powders and the porosity of the prepared sample. The XRD pattern (Fig. 1e) shows predominantly the Al_5_C_3_N phase. The diffraction peaks correspond to the planes of Al_5_C_3_N, confirming the hexagonal P6_3_mc space group (No. 186). The Rietveld refinement of the XRD data (Fig. 1f) demonstrates a high degree of correlation between the calculated and observed patterns, with the cell parameters *a* = 3.279 Å and *c* = 21.581 Å, consistent with previously reported findings in other studies^20^.

### Chemical exfoliation and formation of AlN nanosheets

**Fig. 2:**
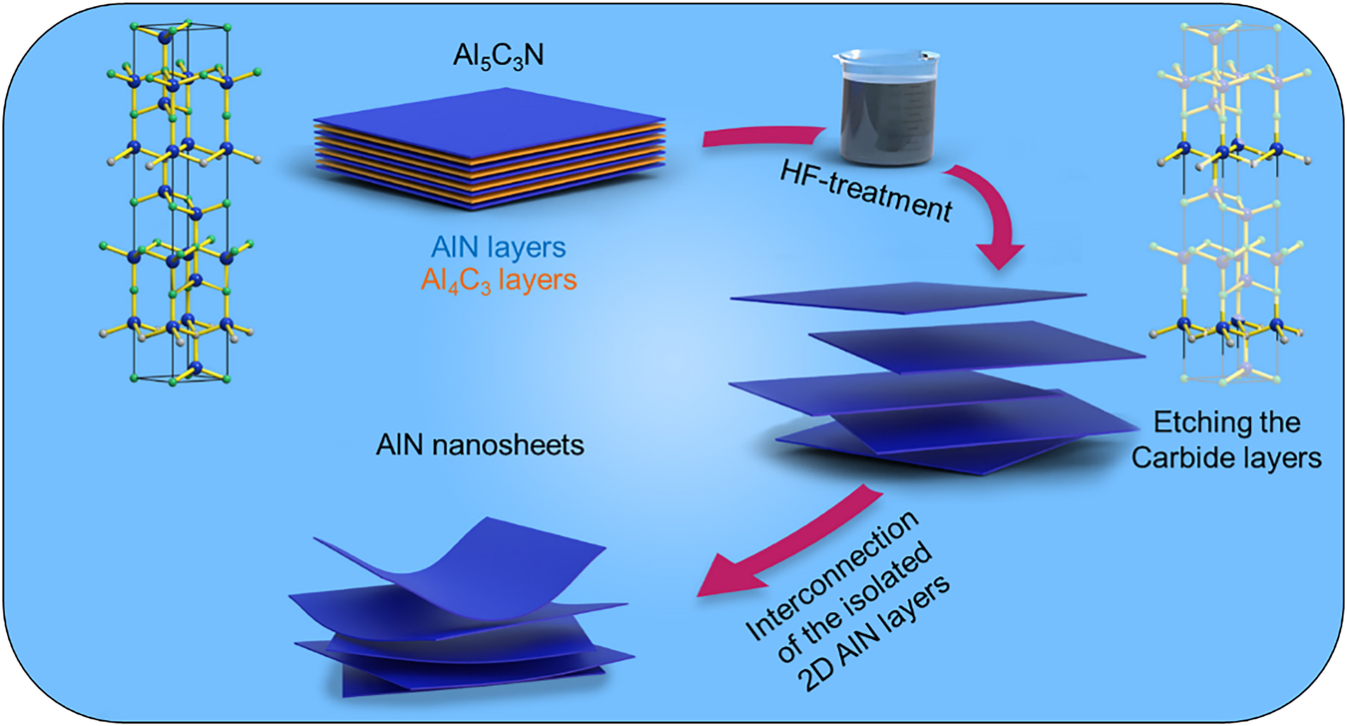
Schematic illustration of AlN nanosheet formation via chemical exfoliation of Al₅C₃N.

Once the precursor is synthesized, the next objective is the chemical etching, taking advantage of its layered structure and chemical configuration. The relative strength of the chemical bonds of Al_5_C_3_N was predicted by Xu et al.^19^, showing that Al_5_C_3_N contains a hexagonal AlN slab and an arrangement of Al and C atoms similar to Al_4_C_3_. The covalent bonding states of Al and N are found at lower energy levels (around −7.5 eV and −6.5 eV for Al 3s–N 2p interactions), indicating stronger and more stable bonds. Additionally, Al-N bonds have multiple pp-derived hybridization states at various lower energy peaks, further reinforcing their strength. The differences in bond strength within the layered structure of Al_5_C_3_N can be leveraged for etching the carbide layers in an acidic medium to extract the AlN layers. The graphical illustration of the etching process, depicted in Fig. 2, involves the chemical exfoliation of Al₅C₃N using HF to selectively break Al-C bonds, producing isolated 2D AlN that subsequently interconnect each other to form AlN nanosheets.

**Fig. 3:**
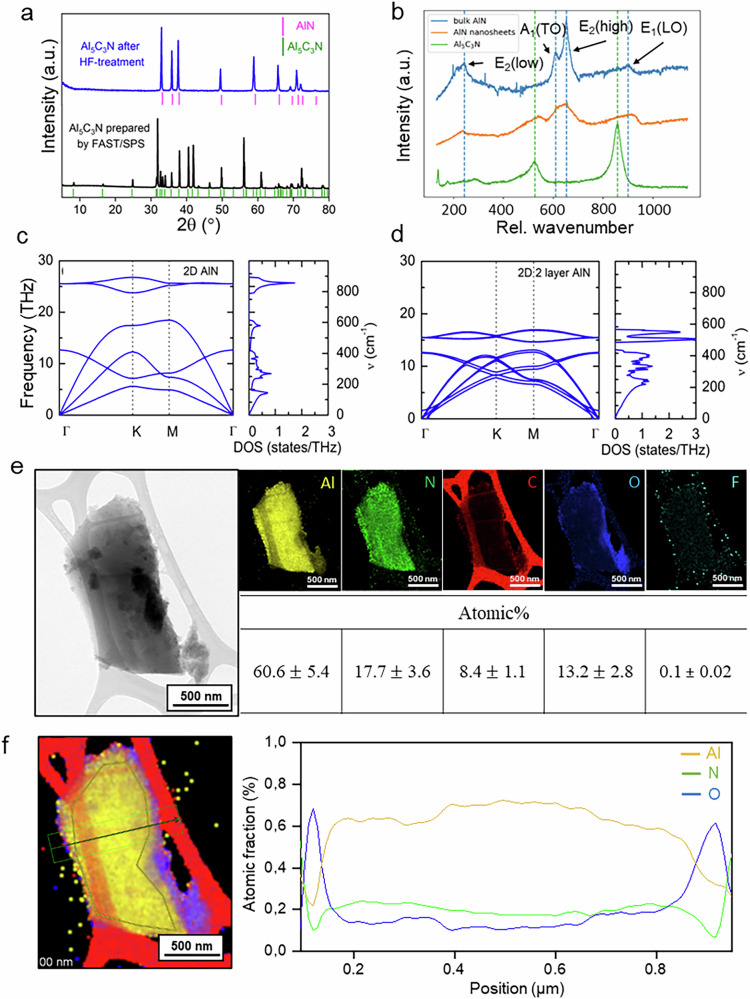
Structural and spectroscopic characterization of AlN nanosheets. **a** XRD pattern of Al₅C₃N before and after HF treatment. **b** Raman spectra of Al₅C₃N (green), AlN nanosheets (orange), and bulk AlN (blue). **c** Phonon band structure and density of states for a single-layer 2D AlN. **d** Phonon band structure and density of states for double-layer 2D AlN. **e** TEM image with corresponding elemental mapping and composition. **f** STEM bright-field image and atomic fraction profile of Al, N, and O in the obtained AlN nanosheets.

The XRD analysis of the etched powder treated in concentrated HF solution, compared with the precursors (Fig. 3a), reveals a significant reduction in the peak intensities associated with the parent Al₅C₃N, leaving AlN-related peaks. The Rietveld refinement analysis (Supplementary Fig. 1a) indicates that the cell parameters of the synthesized AlN (*a* = 3.156 Å and *c* = 5.024 Å) are larger than those of the reported unit cell of bulk AlN (*a* = 3.111 Å and *c* = 4.975 Å)^16^. The XRD pattern thus shows a downshift in the diffraction peaks, with the (002) plane appearing at 2*θ* = 35.357° for the AlN prepared after HF treatment compared to 2*θ* = 35.554° for the reported bulk AlN in the database. This downshift is due to an enlarged c lattice parameter and indicates an increase in the interlayer spacing during the etching process, similar to MXenes^22^. However, in contrast with most HF-etched MXenes, we observed a lower degree of downshift of the (002) peak that can likely be attributed to the selective etching of Al_4_C_3_ slabs, which are thicker than the A-layers in MAX phases, similar to the reported Hf_3_C_2_T_x_ MXene, where thick [Al(Si)]C slabs were etched from Hf_3_[Al(Si)]_4_C_6_ MAX precursor^23^. In addition to the differences in peak intensities compared to bulk AlN, all these results hint at the imperfect reassembling of isolated 2D AlN into more stable multilayered AlN nanosheets after the exfoliation process, with these layers potentially aligning with random orientations.

The Raman backscattering spectra (Fig. 3b) of Al_5_C_3_N, show two prominent peaks at 525 and 855 cm^-1^, which are characteristic of the hexagonal starting material. The exfoliation process results in a pronounced change of the phonon spectrum with five phonon modes appearing at 288, 541, 618, 654, and 916 cm^-1^. Except for the peak at 541 cm^-1^, four phonon modes can be attributed to the E_2_(low), A_1_(TO), E_2_(high), and E_1_(LO) of hexagonal bulk AlN^24^. The small shifts of the E_2_(low) and E_1_(LO) modes indicate the presence of lattice strain that might be caused by the imperfect stacking of interconnected 2D AlN sheets or the presence of impurities. The presence of these modes suggests that the exfoliation process did not result in the formation of pure 2D AlN layers, but rather multilayer AlN nanosheets.

The phonon mode located at 541 cm^-1^ can be attributed to different origins. (i) The etching process was incomplete and left behind some residual Al_5_C_3_N. The small shift of this mode compared to Al_5_C_3_N might be related to lattice distortion due to surface terminations, and localized defects were introduced during etching. (ii) The phonon mode at 541 cm^-1^ is characteristic of stacked AlN nanosheets. This is consistent with our ab-initio calculations (Fig. 3c, d) that show a split LO phonon mode for an AlN double layer with peaks located at 507 and 550 cm^-1^. With an increasing number of stacked AlN layers, the split of the LO phonon disappears, and the mode becomes degenerate. It is conceivable that both explanations may contribute to the phonon mode observed at 541 cm^-1^.

The SEM image (Supplementary Fig. 1b) after exfoliation of Al_5_C_3_N in HF shows the multilayered morphology, similar to other early-stage chemically exfoliated materials such as MXenes^8,25^. The TEM image (Fig. 3e) shows an electron-transparent flake, and the EDS elemental mapping of the flakes indicates the presence of Al, N, and O, while C is low (Fig. 3e). This shows that the exfoliated products are AlN-rich rather than pure stoichiometric AlN. The atomic fraction line profile in Fig. 3f also shows that oxygen is predominantly concentrated at the edges of the flakes (the same behavior for fluorine), which are more reactive zones for interaction with the environment and prone to oxidation^26^. Moreover, from the quantification, a nitrogen deficiency is observed, with the Al molar ratio deviating from 1:1, as the amount of Al is approximately triple that of N. The excess Al cannot be fully accounted for within the AlN lattice and is therefore likely present in the form of Al–O or Al–F bonds at the surface. This interpretation is consistent with the detection of oxygen and minor fluorine, and suggests that the nanosheets are best described as AlN-rich layers terminated with –O/–OH and –F functional groups. From the EDS data, the approximate composition can be estimated as AlNₓO_y_F_z_, where *x* ≈ 0.3-0.5, *y* ≈ 0.2-0.3, and *z* ≈ 0.002-0.01, although the exact stoichiometry likely varies across flakes due to heterogeneous oxidation. Furthermore, some residual carbon particles were observed after etching, arising from the interaction of fluoride ions with Al–C bonds that weaken and cleave these bonds, leaving carbon byproducts. However, no carbon-related features were detected in the Raman spectra within the D and G band region, indicating that such residues were largely removed during washing, and are consistent with the “AlN-rich” description.

Atomic force microscopy (AFM) measurements (Supplementary Fig. 2) also confirmed that the thickness of the exfoliated nanosheets lies in the range of tens of nanometers, depending on the flake. The representative flakes showed a height of ~40 nm. These values confirm that the exfoliated products are multilayered AlN nanosheets rather than monolayers.

**Fig. 4:**
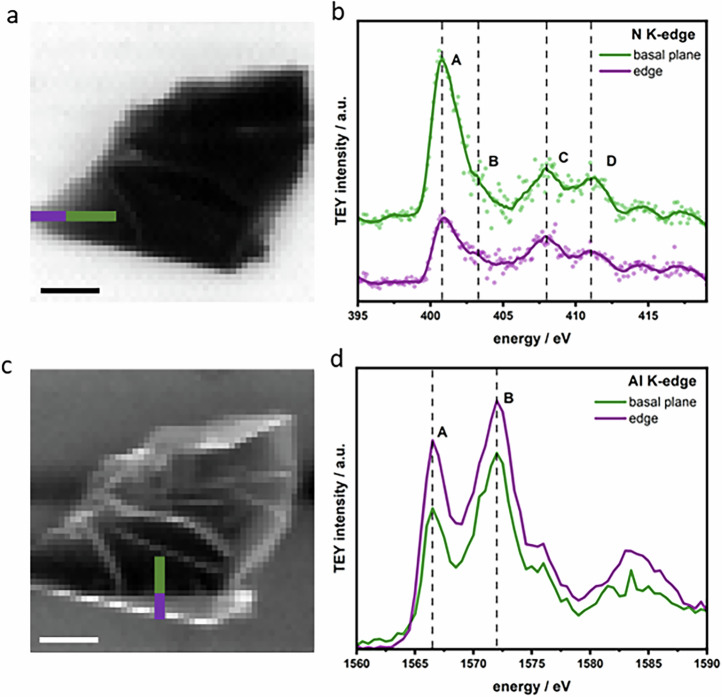
Nanoscale spectromicroscopy of AlN nanosheets. **a** TEY-SXM image at the N K-edge (400 eV); scale bar: 1 μm. **b** N K-edge TEY-XAS measured along the line in (**a**); scatter points represent raw data and solid lines are Savitzky–Golay smoothed spectra. **c** TEY-SXM image at the Al K-edge (1565 eV); scale bar: 1 μm. **d** Al K-edge TEY-XAS spectra measured along the line in (**c**).

Total electron yield scanning X-ray microscopy (TEY-SXM) measurements of the obtained AlN provided critical insights into its surface composition and electronic structure (Fig. 4). The chemical bonding of Al and N atoms is determined by X-ray absorption spectroscopy (XAS) at the respective K-edges acquired on an individual multilayered flake as imaged in Fig. 4a, c. The N K-edge XAS spectra (Fig. 4b) display four prominent features at 400.5, 403.2, 408, and 411.2 eV, labeled as peaks A, B, C, and D, respectively. Similar features have been reported for bulk transition metal nitrides (CrN and TiN)^27^ and AlN^28^, where peaks A and B are attributed to electronic transitions into unoccupied hybridized states involving N 2p and metal 3 d orbitals, while peaks C and D correspond to transitions to unoccupied N 2p states hybridized with metal 4 s orbitals. Despite the similarity in features, the chemical shift of peaks C and D compared to peak A on AlN nanosheets is +7.0 and +10.5 eV compared to +5.8 and +10.1 eV for bulk AlN, suggesting a distinct bonding environment for N atoms in AlN nanosheets compared to bulk AlN. The relative intensities of peaks A, B, and C also indicate a different chemistry between the edge and the basal plane of the flake, which is also observed in the case of MXenes, due to the relatively higher oxidation of the edge after the etching process^29^. The Al K-edge XAS spectra (Fig. 4d) show two distinct features A and B at 1566 and 1572 eV, with a chemical shift of +5.5 eV, which is similar to bulk AlN ( + 5.6 eV), indicating similar bonding of Al in AlN nanosheets as for bulk AlN^30^.

**Fig. 5:**
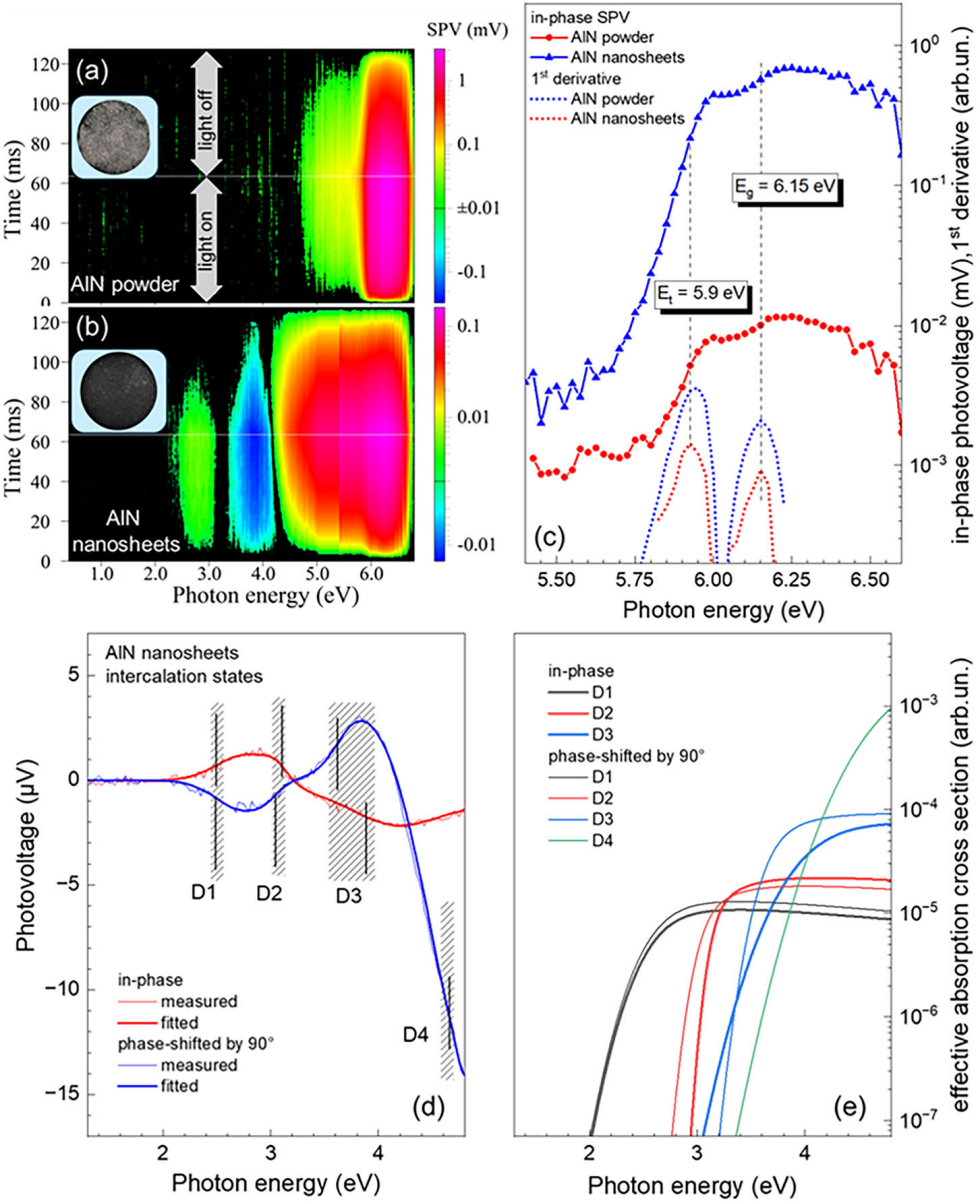
Surface photovoltage (SPV) characterization of AlN nanosheets. **a** Contour plots of the modulated transient SPV signals for the AlN reference (samples mounted on a 10 mm carbon disc). **b** Contour plots for the AlN nanosheets over the full measurement range. Insets in (a) and (b) show the investigated samples consisting of powders fixed on a carbon pad on an ITO glass carrier. **c** Spectra of the in-phase SPV signals and their first derivatives for the AlN reference (blue triangles and lines) and AlN nanosheets (red circles and lines) in the region around the AlN bandgap. The dashed black line indicates the bulk AlN bandgap; additional black markers at 6.15 and 5.9 eV highlight electronic states at particle/nanosheet edges. **d** Spectra of the measured (thin lines) and fitted (thick lines) in-phase (red) and 90° phase-shifted (blue) SPV signals in the region of deep defect states in AlN nanosheets. **e** Effective absorption cross sections of deep defect transition states (D1–D4), used for fitting the in-phase (thick lines) and 90° phase-shifted (thin lines) SPV spectra (black, red, blue, and green curves, respectively). The hatched regions at 2.5, 3.0–3.11, 3.6–3.87, and 4.65 eV correspond to D1–D4 transition energies due to intercalation in AlN nanosheets.

Surface photovoltage (SPV) measurements, as shown in Fig. 5, present the contour plots of modulated transient SPV signals for both chemically etched AlN nanosheets derived from Al₅C₃N (b) and the AlN reference (a) across the full measurement range of photon energy. For the AlN reference, positive SPV signals emerge at approximately 4.5 eV and increase steeply around 5.7 eV, significantly below the AlN bandgap (Eg = 6.12 eV^31^). Due to the notably weaker SPV signals in the chemically exfoliated AlN nanosheets, measurements were performed with higher averaging. Weak positive and negative SPV signals were observed between 2.4–3.1 eV and 3.4–4.2 eV, respectively, while relatively strong positive SPV signals appeared around 4.2 eV. Figure 5c displays spectra of in-phase SPV signals and their first derivatives near the bandgap region, revealing a common inflection point at 6.15 eV for both samples, consistent with the bandgap, and a second inflection point at 5.9 eV, likely associated with defect states. However, deep defect states were prominent in the chemically exfoliated AlN nanosheets, indicating distinct electronic properties compared to reference AlN. Figure 5d illustrates in-phase (x) and 90° phase-shifted (y) SPV spectra for the AlN nanosheets. For small SPV signals with linear intensity dependence and independent superimposed contributions, effective absorption cross sections were fitted, considering the photon flux spectrum (Fig. 5e). Identified transitions, labeled as D1, D2, D3, and D4, correspond to absorption energies of 2.5 (x), 2.5 (y); 3.11 (x), 3.0 (y); 3.87 (x), 3.6 (y); and 4.65 (y) eV, respectively. The results indicate that the synthesized AlN nanosheets exhibit a high density of defects, consistent with the etching process and imperfect stacking of the 2D layers. These defects likely contribute to the observed shifts in Raman peaks and the altered nitrogen bonding environment discussed in the SXM analysis. Notably, the presence of tunable defects offers opportunities to tailor the electronic and chemical properties of AlN nanosheets, particularly enhancing their potential for catalytic applications.

Having thoroughly characterized the synthesized AlN nanosheets, the following mechanism can be proposed for the etching of Al_5_C_3_N into AlN nanosheets. When Al_5_C_3_N is dissolved in HF, the spontaneous dissociation of HF leads to the breaking of the Al-C bonds, resulting in AlN nanosheets with the formation of aluminum fluoride and hydrogen gas following the reaction:

1 $${{{{\rm{Al}}}}}_{5}{{{{\rm{C}}}}}_{3}{{{\rm{N}}}}+12{{{\rm{HF}}}}\to {{{\rm{AlN}}}}+3{{{\rm{C}}}}+{4{{{\rm{AlF}}}}}_{3}+{6{{{\rm{H}}}}}_{2}$$

The AlF₃ formed as a byproduct precipitates during etching and is subsequently dissolved and removed by hot-water washing^32^, which may partly contribute to the reduced yield of AlN nanosheets.

Although this process would suggest the formation of the single layer 2D AlN, a change of the phonon bands from the Raman signature of the AlN nanosheet would be expected from the transition from sp^3^ to sp^2^ bonding configuration. However, the phonon dispersion of the AlN nanosheets resembles that of bulk AlN (Fig. 3c), as previously reported by Davydov et al.^33^. This is consistent with DFT calculations, which show that the phonon modes of monolayer AlN would exhibit a significant redshift compared to multilayer structures, indicating that the experimentally observed nanosheets are not monolayers. On the other hand, a chemical shift of the nitrogen bonds in the XAS N K-edge is detected (Fig. 4a), suggesting that N atoms have a different chemical bonding environment compared to bulk AlN.

We hypothesize that AlN 2D layers reconstruct in multilayered AlN nanosheets after etching of the Al_4_C_3_ phase, due to their lower stability compared to the 3D phase. The driving forces include residual interlayer attractions, the polar nature of Al–N bonds, and the absence of intercalants or surfactants to prevent restacking. Moreover, terminal oxygen and fluorine atoms —particularly at the edges—can create new defect states that are not present in bulk AlN and contribute to sheet–sheet interactions. In future work, the stabilization of true single-atom-layer AlN and the enlargement of flake size may be achieved by introducing surfactants during exfoliation—similar to the approach recently reported to stabilize 2D single-atom gold sheets after wet chemical etching^34^. Further systematic studies will also be required to optimize etching conditions and explore safer synthesis routes, alongside evaluating the oxidation stability and defect formation in AlN nanosheets, in order to advance toward scalable production of true single-layer AlN.

## Conclusions

In summary, we present a facile method for synthesizing AlN nanosheets through the chemical exfoliation of Al_5_C_3_N in HF, offering a potential route for producing 2D AlN. The Al₅C₃N with layered microstructure was initially prepared via the FAST/SPS approach, and then the chemical exfoliation could effectively etch the Al_4_C_3_ slab, resulting in the formation of AlN nanosheets with defects that can be modulated to tailor the properties of the AlN nanosheets for specific applications. This approach can be extended to other aluminum carbonitride family members and Group-III nitride semiconductor materials, paving the way for future exploration of low-dimensional materials. Continued research focusing on optimizing exfoliation techniques and stabilizing single-layer 2D AlN is also needed to unlock the full potential of this material.

## Methods

### Preparation of Al_5_C_3_N

The synthesis of Al_5_C_3_N samples involved the precise combination of aluminum nitride (AlN—Sigma Aldrich, USA, 99 wt% purity; particle size <4 μm) and aluminum carbide (Al_4_C_3_-Sigma Aldrich, USA, 99 wt% purity; particle size <4 μm) powders at a molar ratio of 1:1. Utilizing the FAST/SPS (FCT-HPD5, FCT Systeme GmbH, Germany) methodology, the powders were initially compacted into a 20 mm dia pellet under a uniaxial press at 40 kN for 60 s. Subsequently, the compacted pellet was sintered in a graphite die under vacuum conditions (3 Pa) with a heating rate of 50 °C/min up to 1100 °C, followed by 20 °C/min up to 1800 °C, while maintaining a constant uniaxial pressure of 10 MPa throughout the thermal cycle. Additionally, an isothermal holding time of 30 min ensured the completion of the sintering process, while a spacer ring prevented direct pressure on the sample during sintering, optimizing the final Al_5_C_3_N sample synthesis and structure.

### Preparation of AlN nanosheets

For the preparation of AlN nanosheets, 0.5 g of the as-prepared Al_5_C_3_N powder was gradually dissolved in 10 ml of a 40 wt.% HF solution for 20 min. The reaction was carried out in a 50 mL PTFE reaction flask under stirring through the aid of a magnetic bar with a rotation speed of 500 rpm for 24 h at room temperature. After the reaction had finished, the color of the suspension changed from brown to gray. Afterward, the etching product was washed several times with deionized water and hot deionized water ($\sim$70 °C), followed by centrifugation at 3500 rpm until a neutral pH was achieved. The resultant powder was dispersed in 20 ml DI water, sonicated for 30 min, and then centrifuged at 3500 rpm for 30 min. Finally, the resulting AlN was collected after being dried under vacuum at room temperature for 24 h.

### Characterizations

The microstructure and elemental composition of the Al_5_C_3_N precursor and etched material were examined by a scanning electron microscope (SEM, GeminiSEM 500, Zeiss, Germany) with energy dispersive X-ray (EDS) spectroscopy. TEM analysis was conducted with a Titan Themis 200 from Thermofisher Scientific (formerly FEI) set at 200 kV, and the chemical composition was measured with EDS using a SuperX detector G2. The sample preparation was executed by drop-casting the solution on a lacey carbon grid that was subsequently allowed to dry at room temperature.

The structural characteristics of the precursor and resulting etched material were analyzed by X-ray diffraction (XRD) using an X-ray diffractometer (D8 Advance, Bruker, Germany) with Cu Kα radiation (*λ* = 1.54 Å). The data were collected in the 2θ range of 5–80° with a 0.02° step size and 1 s/step. Data analysis was conducted using the HighScore Plus 4.9 software package.

The crystalline structure of the synthesized AlN nanosheets and commercial bulk AlN powder (Sigma-Aldrich, 99% purity, particle size <4μm) was investigated by Raman scattering in backscattering geometry. A 457 nm solid-state laser was used as the excitation source, and all measurements were conducted at room temperature.

AFM measurements were performed on a Park Systems XE-100 in non-contact mode under ambient conditions. AlN nanosheets were briefly sonicated in isopropanol and drop-cast onto pre-cleaned Si wafers. The images were processed for background and tilt correction.

Scanning X-ray spectromicroscopy was carried out at the MAXYMUS end station^35^ at the BESSY II electron storage ring operated by the Helmholtz-Zentrum Berlin für Materialien und Energie. These measurements were carried out in a vacuum, utilizing the total electron yield (TEY) detection mode. Monochromatic x-rays are focused onto the sample (spot size ~25 nm) by means of both a diffractive lens [Fresnel zone plate (FZP)] and a pinhole [order selective aperture (OSA)]. Proportional to the local x-ray absorption, secondary electrons are emitted from the sample surface region to the biased (+ 90 V) OSA. The current flowing to the sample, replacing these emitted electrons, is amplified, converted into pulses, and thereby precisely measured while raster scanning the sample yields a 2D image of local x-ray absorption. Recording images at a range of incident photon energies (2D energy stacks) provides local x-ray absorption spectra, here for both the N and Al K-edges. For these measurements, the AlN flakes were simply dispersed in isopropanol and drop-cast onto a gold-coated Si wafer.

For testing electronic transitions in the AlN nanosheets prepared by chemical etching, modulated transient surface photovoltage (SPV)^36,37^ measurements were compared with those on a reference AlN powder (Sigma Aldrich, USA, 99 wt% purity; particle size <4 μm). Incidentally, highly sensitive SPV techniques were recently developed for the analysis of electronic transitions over the entire bandgap of ultra-wide bandgap materials such as diamond, Ga_2_O_3,_ or AlN^38^. For modulated illumination, a laser-driven light source (EQ99-X, Hamamatsu) with a mirrorless double-prism monochromator based on fused silica (DPM100, Freiberg Instruments) was used. The modulation frequency was set to 8 Hz. The analysis following the effective absorption cross section was performed with regard to the reported tutorial and algorithm^39,40^.

### Computational details

The phonon band structures and density of states were obtained from a first-principles approach using density functional theory (DFT). AlN was modeled in a slab geometry with a vacuum region of 24 Å. For the calculations, the Vienna ab initio simulation package (VASP)^41,42^ was used employing the generalized gradient approximation. A plane wave cutoff of 800 eV and a Γ-centered k-point mesh of 13×13×1 was used. Ionic relaxation of the atoms in the supercells was carried out until residual forces reached a value of ≤10^-3 ^eV/Å. For all calculations, Van der Waals interactions were taken into account using the formalism according to Klimes, Bowler, and Michelides^43,44^. The phonon band-structure and density-of-states were obtained using the program package phonopy^45,46^.

## Supplementary information

Chemical Exfoliation of Layered Al₅C₃N for the Synthesis of AlN Nanosheets

## Data Availability

The data that support the findings of this study are available from the corresponding authors upon reasonable request.

## References

[1] Novoselov, K. S. et al. Electric field effect in atomically thin carbon films. *Science* **306**, 666–669 (2004). 15499015 10.1126/science.1102896

[2] Zhang, H. Ultrathin two-dimensional nanomaterials. *ACS Nano* **9**, 9451–9469 (2015). 26407037 10.1021/acsnano.5b05040

[3] Geim, A. K. & Grigorieva, I. V. Van Der Waals heterostructures. *Nature* **499**, 419–425 (2013). 23887427 10.1038/nature12385

[4] Cahangirov, S., Topsakal, M., Aktürk, H. & Ciraci, S. Two- and one-dimensional honeycomb structures of silicon and germanium. *Phys. Rev. Lett.* **102**, 236804 (2009). 19658958 10.1103/PhysRevLett.102.236804

[5] Li, L. et al. Black phosphorus field-effect transistors. *Nat. Nanotechnol.* **9**, 372–377 (2014). 24584274 10.1038/nnano.2014.35

[6] Pacilé, D., Meyer, J. C., Girit, ÇÖ & Zettl, A. The two-dimensional phase of boron nitride: few-atomic-layer sheets and suspended membranes. *Appl. Phys. Lett.* **92**, 133107 (2008).

[7] Wang, Q. H., Kalantar-Zadeh, K., Kis, A., Coleman, J. N. & Strano, M. S. Electronics and optoelectronics of two-dimensional transition metal dichalcogenides. *Nat. Nanotechnol.* **7**, 699–712 (2012). 23132225 10.1038/nnano.2012.193

[8] Naguib, M. et al. Two-dimensional nanocrystals produced by exfoliation of Ti_3_AlC_2_. *Adv. Mater*. **23**, 4248–4253 (2011). 10.1002/adma.201102306 21861270

[9] Wang, Z. et al. Two-dimensional wide band-gap nitride semiconductor GaN and AlN materials: properties, fabrication and applications. *J. Mater. Chem. C.* **9**, 17201–17232 (2021).

[10] Beshkova, M. & Yakimova, R. Properties and potential applications of two-dimensional AlN. *Vacuum* **176**, 109231 (2020).

[11] Ding, Y. & Wang, Y. Enhanced piezoelectricity and half-metallicity of fluorinated AlN nanosheets and nanoribbons: a first-principles study. *J. Mater. Chem. C.* **4**, 1517–1526 (2016).

[12] Yadav, V. K., Mir, S. H. & Singh, J. K. Electronic properties and superior CO_2_ capture selectivity of metal nitride (XN) and phosphide (XP) (X = Al, Ga and In) sheets. *Appl. Surf. Sci.* **527**, 146445 (2020).

[13] Wang, Y. et al. A first-principles study of gas adsorption on monolayer AlN sheet. *Vacuum* **147**, 18–23 (2018).

[14] Jia, K. & Luo, X. Adsorption behavior of CO2 molecule on aln and silicene—application to gas capture devices. *PeerJ Mat. Sci.* **2**, e3 (2020).

[15] Zhang, X., Liu, Z. & Hark, S. Synthesis and optical characterization of single-crystalline AlN nanosheets. *Solid State Commun.* **143**, 317–320 (2007).

[16] Tsipas, P. et al. Evidence for graphite-like hexagonal AlN nanosheets epitaxially grown on single crystal Ag (111). *Appl. Phys. Lett.* **103**, 251605-251605-4 (2013).

[17] Geim, A. K. & Novoselov, K. S. The rise of graphene. *Nat. Mater.* **6**, 183–191 (2007). 17330084 10.1038/nmat1849

[18] Jeffrey, G. A. & Wu, V. The structures of the aluminum carbonitrides. *Acta Crystallogr.* **16**, 559–566 (1963).

[19] Xu, X.-W. et al. Ab initio study of the electronic structure and elastic properties of Al_5_C_3_N. *Chin. Phys. B* **20**, 126201 (2011).

[20] Jeffrey, G. A. & Wu, V. The structure of the aluminum carbonitrides. II. *Acta Cryst.* **20**, 538–547 (1966).

[21] Mu, Y., Yu, D. & Wang, M. Combustion synthesis of aluminum carbonitride. *Int. J. Refractory Met. Hard Mater.* **29**, 639–640 (2011).

[22] Alhabeb, M. et al. Guidelines for synthesis and processing of two-dimensional titanium carbide (Ti3C2Tx MXene). *Chem. Mater.* **29**, 7633–7644 (2017).

[23] Zhou, J. et al. Synthesis and electrochemical properties of two-dimensional hafnium carbide. *ACS Nano* **11**, 3841–3850 (2017). 28375599 10.1021/acsnano.7b00030

[24] Fu, J. Q., Song, T. L., Liang, X. X. & Zhao, G. J. First-principle studies of phonons and thermal properties of AlN in wurtzite structure. *J. Phys. Conf. Ser.* **574**, 012046 (2015).

[25] Tang, Q. & Zhou, Z. Graphene-analogous low-dimensional materials. *Prog. Mater. Sci.* **58**, 1244–1315 (2013).

[26] Soomro, R. A., Zhang, P., Fan, B., Wei, Y. & Xu, B. Progression in the oxidation stability of MXenes. *Nano-Micro Lett.* **15**, 108 (2023). 10.1007/s40820-023-01069-7 PMC10113412 37071337

[27] Pandey, N., Gupta, M., Phase, D. M. & Gupta, A. In situ N It K-edge XANES study of iron, cobalt and nickel nitride thin films. *J. Synchrotron Radiat.* **28**, 1504–1510 (2021). 34475297 10.1107/S1600577521006822

[28] Pao, C. W. et al. Electronic structures of group-III–nitride nanorods studied by X-ray absorption, X-ray emission, and Raman spectroscopy. *Appl. Phys. Lett.* **88**, 223113 (2006).

[29] Al-Temimy, A. et al. Spatially resolved X-ray absorption spectroscopy investigation of individual cation-intercalated multi-layered Ti3C2Tx MXene particles. *Appl. Surf. Sci.* **530**, 147157 (2020).

[30] Mogi, M. et al. Theoretical investigation of Al K-edge x-ray absorption spectra of Al, AlN and Al_2_O_3_. *Mater. Trans.* **45**, 2031–2034 (2004).

[31] Li, J. et al. Band structure and fundamental optical transitions in Wurtzite AlN. *Appl. Phys. Lett*. **83**, 5163–5165 (2003).

[32] Liu, S. et al. Mild fabrication of SiC/C nanosheets with prolonged cycling stability as supercapacitor. *J. Mater. Sci. Technol.* **110**, 178–186 (2022).

[33] Davydov, V. Y. U. et al. Phonon dispersion and Raman scattering in hexagonal GaN and AlN. *Phys. Rev. B* **58**, 12899–12907 (1998).

[34] Kashiwaya, S. et al. Synthesis of goldene comprising single-atom layer gold. *Nat. Synth.* **3**, 744–751 (2024).

[35] Weigand, M. et al. TimeMaxyne: a shot-noise limited, time-resolved pump-and-probe acquisition system capable of 50 GHz frequencies for synchrotron-based X-ray microscopy. *Crystals* **12**. 10.3390/cryst12081029 (2022).

[36] Kronik, L. & Shapira, Y. Surface photovoltage phenomena: theory, experiment, and applications. *Surf. Sci. Rep.* **37**, 1–206 (1999).

[37] Dittrich, T. & Fengler, S. *Surface Photovoltage Analysis of Photoactive Materials* (World Scientific, 2020).

[38] Dittrich, T. & Fengler, S. Surface photovoltage spectroscopy of ultrawide bandgap materials. *Rapid Res. Lett.* **19**, 2400384 (2025).

[39] Alkauskas, A., McCluskey, M. D. & Van de Walle, C. G. Tutorial: defects in semiconductors—combining experiment and theory. *J. Appl. Phys.* **119** (2016).

[40] Fengler, S., Ponomarev, I. & Dittrich, T. Effective absorption cross sections of defects in high pressure high temperature diamond studied by modulated surface photovoltage spectroscopy. *Appl. Mater. Sci*. e202500531. 10.1002/pssa.202500531 (2025).

[41] Kresse, G. & Furthmüller, J. Efficient iterative schemes for ab initio total-energy calculations using a plane-wave basis set. *Phys. Rev. B* **54**, 11169–11186 (1996). 10.1103/physrevb.54.11169 9984901

[42] Kresse, G. & Joubert, D. From ultrasoft pseudopotentials to the projector augmented-wave method. *Phys. Rev. B* **59**, 1758–1775 (1999).

[43] Klimeš, J., Bowler, D. R. & Michaelides, A. Chemical accuracy for the van Der Waals density functional. *J. Phys.: Condens. Matter* **22**, 022201 (2009). 21386245 10.1088/0953-8984/22/2/022201

[44] Klimeš, J., Bowler, D. R. & Michaelides, A. Van Der Waals density functionals applied to solids. *Phys. Rev. B* **83**, 195131. 10.1103/PhysRevB.83.195131 (2011).

[45] Togo, A., Chaput, L., Tadano, T. & Tanaka, I. Implementation strategies in phonopy and phono3py. *J. Phys. Condens. Matter* **35**, 353001 (2023). 10.1088/1361-648X/acd831 37220761

[46] Togo, A. First-principles phonon calculations with phonopy and phonopy. *J. Phys. Soc. Jpn.* **92**, 012001 (2023).

