# Supplementary material for: Chemical exfoliation of layered Al5C3N for the synthesis of AlN nanosheets
Source: Commun Mater. 2025 Dec 8;7(1):2. doi: 10.1038/s43246-025-01019-3 (PMC12764427; doi:10.1038/s43246-025-01019-3)
Supplement: Supplementary file 1 — Chemical Exfoliation of Layered Al₅C₃N for the Synthesis of AlN Nanosheets [file 43246_2025_1019_MOESM1_ESM.docx]

**Chemical Exfoliation of Layered Al₅C₃N for the Synthesis of AlN Nanosheets**

Nima Amousa,^a*^ Melina Poll,^b^ Louis Godeffroy,^a^ Pedro Berastegui,^c^ Norbert H. Nickel,^a^ Namrata Sharma,^a,d^ Olivier Donzel-Gargand,^e^ Thomas Dittrich,^a^ Steffen Fengler,^f^ Sebastian Wintz,^g^ Tristan Petit,^a^ Ulf Jansson,^c^ Jesus Gonzalez-Julian^h*^

*a Nanoscale Solid−Liquid Interfaces, Helmholtz-Zentrum Berlin für Materialien und Energie GmbH, Schwarzschildstr. 8, 12489 Berlin, Germany*

*b Institute of Energy Materials and Devices (IMD-2): Materials Synthesis and Processing, Forschungszentrum Jülich GmbH, 52425, Jülich, Germany*

*c Department of Chemistry-Ångström, Uppsala University Box 538, SE-751 21, Uppsala, Sweden*

*d Faculty of Mathematics and Natural Sciences, TU Berlin, Hardenbergstraße 36, 10623 Berlin, Germany*

*e Department of Material Science and Engineering, Ångström laboratory, Uppsala University, Uppsala, Sweden*

*f Freiberg Instrument GmbH, Delfter Str. 6, 09599 Freiberg, Germany*

*g Institut für Nanospektroskopie, Helmholtz-Zentrum Berlin für Materialien und Energie GmbH, 12489 Berlin, Germany*

*h Laboratory of Thermo-Structural Composites (LCTS), 3 Allée La Boétie, F 33600, Pessac, France*

********Corresponding authors:* [*gonzalez@lcts.u-bordeaux.fr*](mailto:gonzalez@lcts.u-bordeaux.fr) *and* [*nima.amousa@helmholtz-berlin.de*](mailto:nima.amousa@helmholtz-berlin.de)


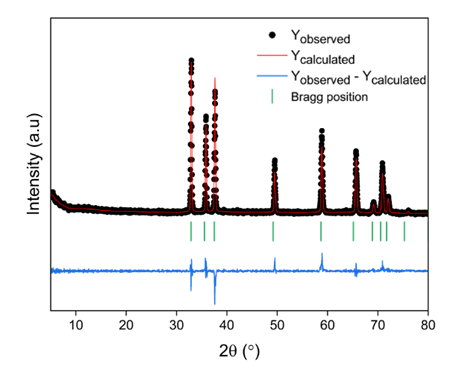


a

b


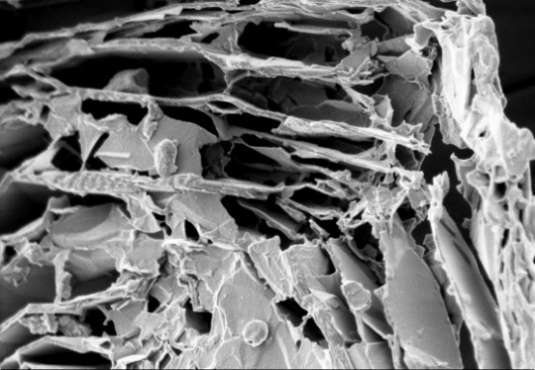


**2 μm**

Supplementary Figure S1. (a) Rietveld refinement and (b) SEM image of the obtained AlN nanosheets.

a


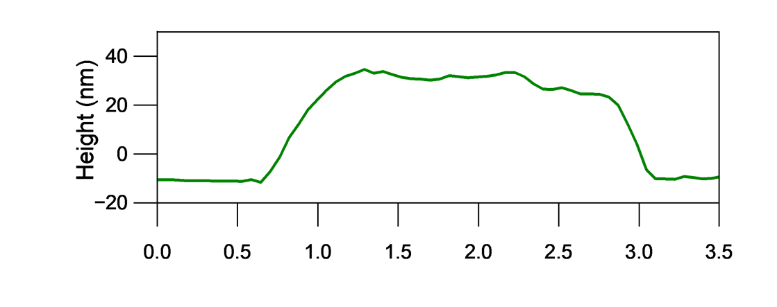

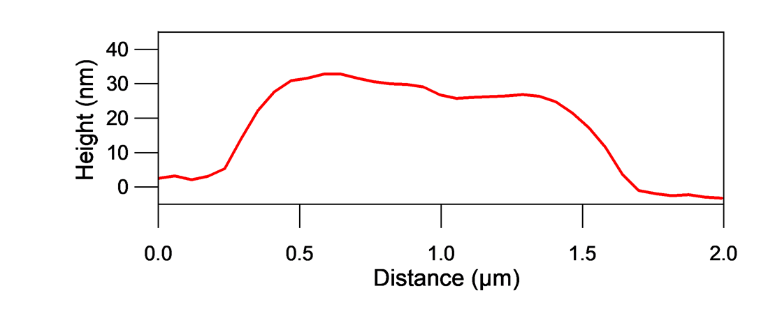

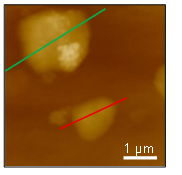


b

c

Supplementary Figure S2. (a) AFM topography image of AlN nanosheets deposited on a Si wafer substrate. (b and c) Corresponding height profiles along the green and red scan lines.
